# A graphite foreign body granuloma that caused palatal perforation. Case report and literature review

**DOI:** 10.4317/jced.61474

**Published:** 2024-05-01

**Authors:** Athina Tosiou, Eleni-Marina Kalogirou, Dimitrios Vlachodimitropoulos, Konstantinos I. Tosios, Vasileios Petsinis

**Affiliations:** 1 Senior student, Dental School, University of Athens, Athens, Greece; 2 DDS, MSc, PhD. Faculty of Health and Rehabilitation Sciences, Metropolitan College, Athens, Greece; 3 MD, PhD. Professor, Department of Forensics and Toxicology, Medical School, University of Athens, Athens, Greece; 4 DDS, PhD. Associate Professor, Department of Oral Medicine & Pathology and Hospital Dentistry, Dental School, University of Athens, Athens, Greece; 5 DDS, MD, PhD. Assistant Professor, Department of Oral and Maxillofacial Surgery, Dental School, University of Athens, Athens, Greece

## Abstract

**Background:**

We present an unusual case of a graphite foreign body granuloma causing palatal perforation.

**Case description:**

A 62-year-old female presented with a macule on the hard palate clinically consistent with a blue nevus. On biopsy a black nodular mass was excised, establishing oroantral communication that was verified by a computed tomography scan. A diagnosis of malignant melanoma was strongly suspected, but microscopic examination showed a graphite foreign body granuloma. It was suggested that the graphite was implanted in a thin area of the palatal bone causing perforation.

**Conclusions:**

Graphite tattoos should be excised, both for diagnostics purposes and the possibility of causing tissue destruction by generating a foreign body granuloma reaction.

** Key words:**Pencil core granuloma, graphite, foreign body, palate, case report.

## Introduction

A penetrating injury from a “lead” pencil may cause implantation of its core in the tissues. Degradation of the “lead” may release graphite, a crystalline form of elemental carbon, generating a blue-gray macular graphite tattoo and occasionally a foreign body granulomatous reaction known as pencil-core granuloma or graphite foreign body granuloma (1). Graphite foreign body granulomas have been mostly described in the skin (1,2) and eye (3), where they may manifest as tumor-like lesions simulating malignant melanoma.

Graphite is considered the second most common exogenous pigment deposited in the oral mucosa following amalgam (4), the latter accounting for most of solitary pigmented lesions of the oral mucosa (5). In contrast to an amalgam tattoo, where history combined with clinical and radiographic information may suggest the diagnosis, documentation of a graphite tattoo may be challenging in patients not recalling previous trauma with a pencil, and requires biopsy (6-8). A review of the pertinent literature disclosed eight documented cases of graphite tattoo in the oral soft tissues (4,6-12), four of them consistent with graphite foreign body granuloma (4,6,8,10).

We present an unusual case of a graphite foreign body granuloma that caused palatal perforation and review the literature on graphite deposition in the oral mucosa.

Case Report

A 62-year-old female was referred for diagnosis and management of a macule on the hard palate incidentally noticed by her dentist during fabrication of a fixed prosthesis, approximately 3 weeks before presentation. The patient was unaware of its presence, although she had been subjected to numerous dental examinations and procedures in the past years by other dentists. She was medicated with levothyroxine for hypothyroidism and had been smoking 5-6 cigarettes per day for the past 35 years.

**Figure 1:**
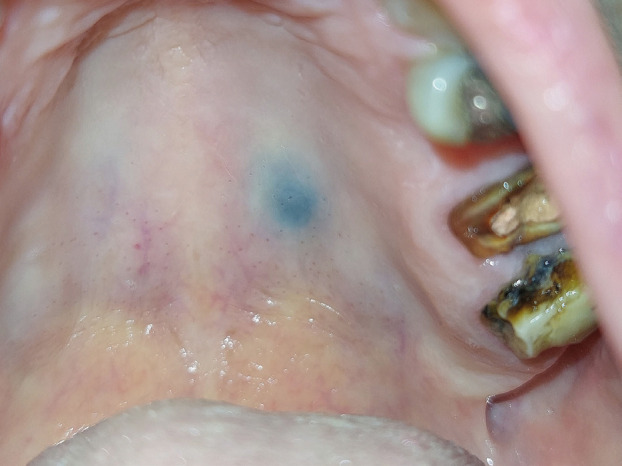
Clinical examination shows a bluish macule on the hard palate, left to the midline, with normal surface and fading outline, consistent with a blue nevus.

Clinical examination disclosed a small (<0.6 cm), solitary, pigmented macule on the hard palate (Fig. 1). It was symmetric and had a homogenous blue color, fading but discrete borders, and smooth surface. When digital pressure was applied the lesions did not blanch and the patient did not report discomfort. One of the nearby teeth had a restoration with dental amalgam. The rest of the oral mucosa was within normal limits and no lymph nodes were palpated in the neck. The patient could not recall an injury in the area.

**Figure 2:**
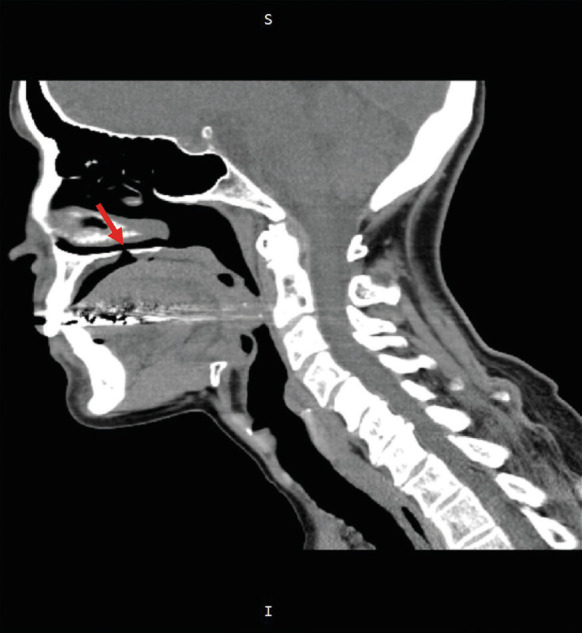
CT scan shows the oroantral communication (arrow). No other pathological features are seen in the oral or nasal mucosa.

With the clinical diagnosis of a blue nevus, an excisional biopsy was carried out under local infiltration anesthesia. During biopsy a black nodular mass that extended through a palatal deficit towards the nasal cavity was excised. A Valsalva maneuver was negative for oronasal communication, but a few hours later the patient reported that water was “pouring from the nose”. Based on those features, a malignant melanoma, possibly a primary sinonasal melanoma originating from the respiratory mucosa (13) was considered. However, a computed tomography (CT) scan that confirmed the presence of oroantral communication did not reveal signs suggestive of a malignant melanoma (Fig. 2).

**Figure 3:**
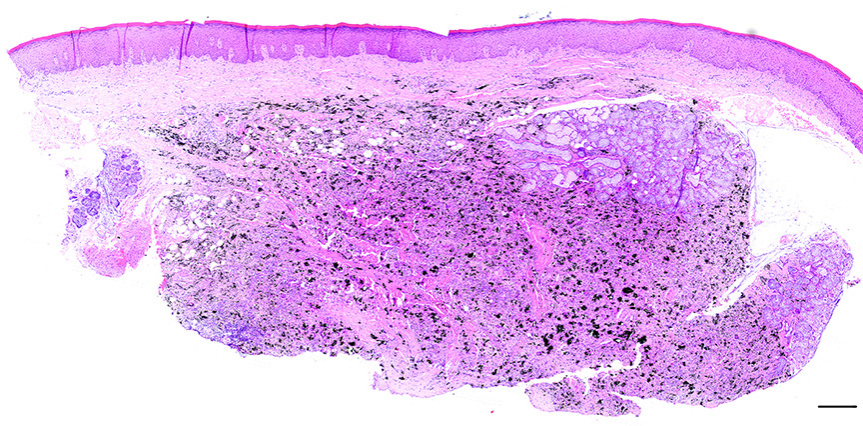
Microscopic examination shows particles of a black exogenous material embedded in the connective tissue stroma of the mucosa and salivary glands (hematoxylin and eosin stain, scale bar = 500μm).

**Figure 4:**
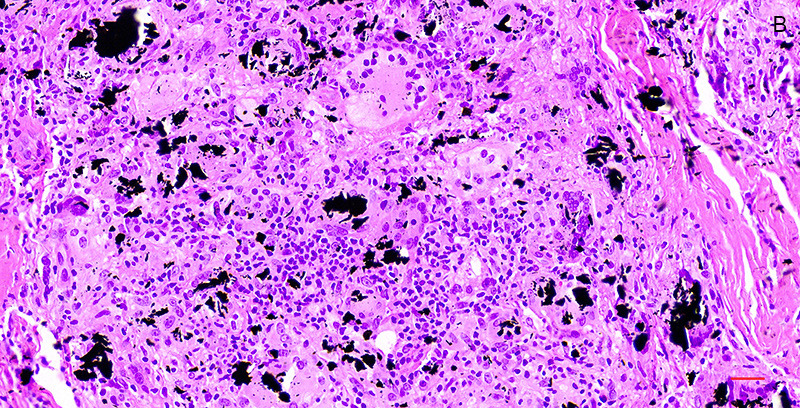
Microscopic examination shows that the black exogenous material is engulfed by epithelioid cells and phagocytosed by multinucleated giant cells of the foreign body type (hematoxylin and eosin stain, scale bar=50μm).

Microscopic examination of 5μm thick, formalin-fixed and paraffin-embedded tissue sections stained with hematoxylin and eosin showed particles of a black exogenous material embedded in the connective tissue of the mucosa and underlying minor salivary glands (Fig. 3). The particles had irregular outlines, and variable size and shape. They were mostly engulfed by epithelioid cells, consistent with macrophages, and occasionally phagocytized by multinucleated giant cells of the foreign body type (Fig. 4). No pigmentation was seen along collagen bundles or the epithelial, vascular, or neural basement membranes, while polarized light examination showed peripheral birefringence of the black particles. The covering mucosa was normal, while the surrounding connective tissue showed perivascular lymphocytic infiltrations. Based on the distribution of the particles, a diagnosis of graphite foreign body granuloma was rendered. The pencil’s core was not found in multiple tissue sections.

Excision of the remaining graphite from the palatal bone and closure of the oronasal communication were successfully performed.

The patient provided informed consent for use of the material for educational and scientific purposes. The guidelines of the Helsinki Declaration were followed in this investigation.

## Discussion

Table 1 summarizes the main features of eight documented cases of oral graphite tattoos and the present one. A case where diagnosis was based on the lack of amalgam restorations in the mouth, but the microscopic description is more consistent with silver deposition (14), and a case where pencil cores were implanted into the maxillary bone but did not cause mucosal discoloration (15), are not included. Eight patients were females, aged 5 to 62 years. The anterior hard palate and the facial maxillary gingiva were the most common locations. All cases appeared as macules measuring 4-15 mm in diameter and were asymptomatic.

The differential diagnosis of a solitary pigmented macule on the oral mucosa includes melanotic lesions and deposition of exogenous materials, as well as vascular lesions. The most common melanotic lesion of the lips and oral mucosa is the melanotic macule (5,16). It is usually found in young females as a small macule of light to dark brown color (5,16). Its preferred intraoral locations are the maxillary gingiva and the palate (16). Oral acquired melanocytic nevi are rare and usually affect young females (5,16,17). Most are of the intramucosal type, but it is the less common blue nevus that has a strong predilection for the hard palate and a characteristic blue color (16,17). Oral melanoacanthoma is an even rarer reactive melanocytic lesion combing epithelial hyperplasia with the pronounced presence of dentritic melanocytes and melanin pigmentation (18). It shows a strong predilection for young African American females but may also appear in Caucasian (18). The palate is the second most common location, following the buccal mucosa where almost half of the cases appear, and although the typical color is dark black or brown, it may occasionally be blue (18). Oral malignant melanoma is a very rare tumor that usually affects older males (13,19). It shows a preponderance for the hard palate and palatal gingiva, where it presents as a macule, a plaque-like lesion, or a nodule that may be asymmetric with irregular borders and a wide range of colors (13). In its early stages it is asymptomatic, may imitate benign melanotic lesions (5) and be an incidental finding (19). Amalgam pigmentation in the form of amalgam tattoo is the commonest oral pigmented lesion (5,16). Most patients are adults (5) and almost half of the lesions are located on the gingiva and alveolar mucosa as flat macules (16). An amalgam tattoo on the hard palate is unusual (16) and may by similar to a blue nevus (17). Finally, oral Kaposi sarcoma (KS) usually develops in the hard and soft palate, and in its early stages it may appear as an asymptomatic small macule with variegated color that does not blanch on pressure (20). It is more common in the epidemic or acquired immunodeﬁciency syndrome (AIDS)-associated KS, where the oral mucosa is the initial site of clinical disease approximately 22%, and unusual in the classic or Mediterranean KS, iatrogenic or post-transplant KS, and endemic or African KS (20). Its recognition in the absence of an indicative medical history would be difficult.

Diagnosis of graphite tattoo is based on history and the exclusion by microscopic examination of other exogenous pigments, in particular amalgam. In contrast to an amalgam tattoo, where history combined with clinical and radiographic information may suggest the diagnosis, documentation of a graphite tattoo may be challenging in patients not recalling previous trauma with a pencil and usually requires biopsy (6-8). Only three patients could recall injury from a pencil, 2 (12), 12 (4), and 43 (10) years before presentation. As for the lesion to appear a lengthy process of disintegration of the “lead” before release of graphite is necessary (1), it is reasonable that a trauma that happened many years ago could not remember by our patient. The longest interval between implantation of a pencil core and diagnosis of the lesion was 62 years, in the forehead skin of a 71-year-old female (21). Occasional cutaneous (1) and ocular (3) graphite foreign body granulomas may manifest as tumor-like lesions simulating malignant melanoma, as in the present case.

Microscopically, the characteristic chain-like distribution of granules along collagen bundles, around small vessels, and nerve sheaths, or in the epithelial basement membrane zone of the mucosal epithelium and salivary ducts or acini, is not seen (16). In five lesions, including the present one, a foreign body granuloma reaction was seen (4,6,8,10). However, this is not a discriminatory feature from amalgam tattoo, as it may appear in approximately 1/3 of the later (16). It has been reported that birefringence in polarized light following treatment of tissue sections with 10% ammonium sulfide (11) and incineration of tissue sections (7) may help differentiation from amalgam, while electron dispersive spectrography analysis and scanning electron microscopy may document the presence of graphite (4). In our case, the histologic features were consistent with graphite foreign body granuloma, excluding an amalgam tattoo.

There are reports of bone depression in two graphite foreign body granulomas located on the forehead skin (22,23) and one on the radio-ulnar joint (24), respectively, attributed to inflammation or pressure exerted by the lesion (23). Bone destruction was described in two cases involving the labial cortical bone (8) and the superior lateral orbit (25), respectively. In the latter case, the lytic lesion surrounded a pencil core that was embedded in the bone. In our case, it is suggested that the core was implanted in a thin area of the palatal bone and the foreign body granuloma caused the bone perforation. The core was probably fully degraded over time.

Oral graphite tattoos are considered innocuous lesions, and excision is indicated for diagnostic or esthetic purposes (4,10). The present case, however, indicates that when a graphite foreign body granuloma develops, it may be destructive for the surrounding tissues and requires complete removal, as residual graphite may generate a new granulomatous reaction over time (22).

In conclusion, a graphite tattoo may be included in the differential diagnosis of solitary pigmented lesions of the oral mucosa and should be excised, both for diagnostic purposes and the possibility of causing tissue destruction by generating a foreign body granuloma reaction.

## Figures and Tables

**Table 1:** Main features of eight documented cases of mucosal lesions associated with graphite deposition and the present one.

Reference	Age	Sex	Location	Size (mm)	FRB^3^	Age of trauma	Behavior
Peters & Gardner (11)	55	F^1^	palate	n/a^2^	No	n/a	-
Phillips & Vanchit (10)	17	F	facial maxillary interdental papilla, free and attached gingiva, and alveolar mucosa, canine and lateral incisor	n/a	Yes	5-year-old	-
Rihani et al. 2006 (8)	5	F	facial maxillary attached gingiva, primary central and lateral incisor	15	Yes	No	size increase, bone destruction
Rullo et al. 2013 (9)	5	n/a^2^	facial mandibular mucosa, canine	n/a	No	n/a	size increase
Moraes et al. 2015 (4)	62	F	hard palate	5	Yes	13-year-old	-
Molini et al. (6)	27	F	hard palate	7	Yes	No	-
Yeta et al. (7)	24	F	facial maxillary attached gingiva, central and lateral incisors	5	No	No	-
de Carmago Moraes et al. (12)	7	F	hard palate	4	No	5-year-old	-
present case	62	F	hard palate	8	Yes	No	bone destruction

1F, female, 2n/a, not available, 3FBR, foreign body reaction

## Data Availability

The datasets used and/or analyzed during the current study are available from the corresponding author.
